# Macronutrient Management Effects on Nutrient Accumulation, Partitioning, Remobilization, and Yield of Hybrid Maize Cultivars

**DOI:** 10.3389/fpls.2020.01307

**Published:** 2020-09-02

**Authors:** Krishnendu Ray, Hirak Banerjee, Sudarshan Dutta, Sukamal Sarkar, T. Scott Murrell, Vinod K. Singh, Kaushik Majumdar

**Affiliations:** ^1^Sasya Shyamala Krishi Vigyan Kendra, Ramakrishna Mission Vivekananda Educational and Research Institute, Sonarpur, India; ^2^Bidhan Chandra Krishi Viswavidyalaya, Department of Agronomy, Mohanpur, India; ^3^African Plant Nutrition Institute and Mohammed VI Polytechnic University, Benguérir, Morocco; ^4^Purdue University, Department of Agronomy, West Lafayette, IN, United States; ^5^Division of Agronomy, Indian Council of Agricultural Research–Indian Agricultural Research Institute, New Delhi, India

**Keywords:** NPK, nutrient dynamics, dry matter accumulation, remobilization, corn (maize), grain yield, fertilizer

## Abstract

It is critical to understand nutrient dynamics within different plant parts to correctly fine-tune agronomic advices, and to update breeding programs for increasing nutrient use efficiencies and yields. Farmer’s field-based research was conducted to assess the effects of nitrogen (N), phosphorus (P), and potassium (K) levels on dry matter and nutrient accumulation, partitioning, and remobilization dynamics in three popular maize (*Zea mays* L.) hybrids (P3522, P3396, and Rajkumar) over two years in an alluvial soil of West Bengal, India. Experimental results revealed that NPK rates as well as different cultivars significantly (*p* ≤ 0.05) influenced the dry matter accumulation (DMA) in different plant parts of maize at both silking and physiological maturity. The post-silking dry matter accumulation (PSDMA) and post-silking N, P, and K accumulations (PSNA, PSPA, PSKA) were highest in cultivar P3396. However, cultivar P3522 recorded the highest nutrient remobilizations and contributions to grain nutrient content. Total P and K accumulation were highest with 125% of the recommended dose of fertilizer (RDF) while total N accumulation increased even after 150% RDF (100% RDF is 200 kg N, 60 kg P_2_O_5_, and 60 kg K_2_O ha^–1^ for the study region). Application of 125% RDF was optimum for PSDMA. The PSNA continued to increase up to 150% RDF while 125% RDF was optimum for PSPA. Cultivar differences significantly affected both remobilization efficiency (RE) and contribution to grain nutrient content for all tested macronutrients (N, P, and K). In general, RE as well as contribution to grain nutrient content was highest at 125% RDF for N and K, and at 100% RDF for P (either significantly or at par with other rates) for plots receiving nutrients. For all tested cultivars, nutrient remobilization and contribution to grain nutrient content was highest under nutrient-omission plots and absolute control plots. Both year and cultivar effects were non-significant for both grain and stover yields of maize. Application of 75% RDF was sufficient to achieve the attainable yield at the study location. The cultivar P3522 showed higher yield over both P3396 and Rajkumar, irrespective of fertilizer doses, although, the differences were not statistically significant (*p* ≥ 0.05). The study underscores the importance of maize adaptive responses in terms of nutrients accumulation and remobilization at different levels of nutrient availability for stabilizing yield.

## Introduction

Better management practices and seed development programs together have significantly improved maize grain yield during the last century. The breeding efforts supporting yield improvements have resulted in: greater stress tolerance, especially at crowding intensity (Tokatlidis and Koutroubas, 2004); long life of leaves (“stay-green”) (Thomas and Howarth, 2000); efficient root systems (Hammer et al., 2009); greater source activity from ear (female inflorescence which bears grain); increased demand (Tollenaar and Wu, 1999); and augmented individual-plant N accumulation with extensive reproductive-stage buildup (Ciampitti and Vyn, 2013). Stewart et al. (2005) attributed a 57% increase in maize grain yield during 1960 to 2000 to balanced fertilizer inputs (NPK fertilizers and lime).

Plant availability of soil nutrients and genotype are two key factors affecting grain yield and nutrient concentration in maize grain (Ciampitti et al., 2013; Banerjee et al., 2014a; Banerjee et al., 2014b; Banerjee et al., 2015; Dutta et al., 2020). High grain yield also relies on leaf longevity that depends on the balance between post-silking nutrient accumulation and remobilization of vegetative nutrients (Thomas and Howarth, 2000). Maize is very responsive to NPK applications, exhibiting greater whole-plant biomass and grain yield with higher nutrient content. The positive relationship between grain yield and above ground NPK accumulation in whole plant are well established (Setiyono et al., 2010; Ray et al., 2018). Increased fertilizer NPK application rates may lead to an increase in post-silking nutrient accumulation, a delay in leaf senescence, maintenance of accumulation and remobilization of photosynthates, and a concomitant increase in grain yield (Huang et al., 2007). Ciampitti et al. (2013) also found similar variations in grain yield response to fertilizer NPK rates which might be due to differences in soil N supply.

An increase in N supply may decrease the remobilization of vegetative N (at pre-silking stage) to the grain, and therefore does not necessarily increase grain N concentration (Fowler, 2003; Peng et al., 2017). Nitrogen supply at higher than optimum rate for attainable grain yield can increase grain N concentration (Chen et al., 2010; Masoero et al., 2011), but may also increase the amount of N in vegetative organs (Chen et al., 2010; Hou et al., 2012). During breeding of modern stay-green maize cultivars, plants are generally grown under high N inputs in fertile soils (Bertin and Gallais, 2000). Compared with the older senescent hybrids, the stay-green cultivars typically show higher post-silking N accumulation but lower N remobilization (Mi et al., 2003; He et al., 2004; Pommel et al., 2006; Ciampitti and Vyn, 2013). Post-silking photosynthetic activity resulted in greater grain dry matter in maize (Tollenaar et al., 2004; Lee and Tollenaar, 2007), while pre-silking N accumulation *via* vegetative N remobilization during grain filling contributed 45–65% of grain N (Hirel et al., 2007). Phosphorous either alone or in combination with N, plays an important role in plant photosynthesis and dry matter accumulation in maize (Banerjee et al., 2015; Ray et al., 2018). An adequate concentration of P is important for maintaining a high rate of photosynthesis (Marschner, 2012). Deficiency of P causes a greater reduction in carbon fixation and export and consequently alters fixed carbon partitioning (Usuda and Shimogawara, 1991). Greater P applications result in greater leaf area and therefore greater photosynthetically active radiation absorption by the maize canopy, resulting in greater above-ground biomass production (Plénet et al., 2000). In addition, greater N and P remobilization in leaves reduces leaf photosynthesis during grain filling. Little evidence exists for concluding whether or not modern, stay-green maize hybrids accumulate more N and P or if remobilization of leaf N and P prolongs photosynthetic activity during grain filling.

Potassium is a mobile nutrient and moves easily between different organs in plants (Hawkesford et al., 2012). Unlike N and P, only the cationic form (K^+^) exists in plant tissues. It is involved in various metabolic functions, namely enzyme activation, protein synthesis, translocation of soluble metabolites, and maintenance of osmotic balance (Hawkesford et al., 2012). Peng et al. (2012) reported that net K loss before maturity of maize plants brought about significant K accumulation in rhizosphere soil at the end of reproductive growth. After silking, modern maize hybrids take up more N, P, and K than older cultivars (Ning et al., 2013). These relationships demonstrate the complexity of interactions of N, P, and K and reinforce the need for further thorough investigations on the impact of these factors on dynamics of NPK accumulation.

Understanding the complex interactions among dry matter and nutrient accumulation and remobilization, and the grain yield of the crop may help in selecting the hybrids that produce high grain yield with high grain nutrient content that is important for sustainable food and nutrition security (Uribelarrea et al., 2007). Exploring a physiological approach i.e. the dry matter and nutrient accumulation and partitioning at different growth stages can help estimate the dry matter and nutrient dynamics within the plant (Ciampitti et al., 2013).

The joint influences of NPK rates and cultivars on yield, dry matter and nutrient accumulation and partitioning, and remobilization patterns in maize plant components are less explored, especially in the alluvial soils of the humid-tropics—one of the globally major maize growing areas. This documentation is, however, essential for future improvement of grain yield from both agronomic and breeding standpoints. We hypothesized that the dry matter accumulation, nutrient dynamics, and grain yield of maize hybrids vary across growing seasons, cultivars, and rates of NPK-fertilization. In other words, the yield improvement in maize hybrids can be well explained by the pattern of dry matter accumulation and NPK accumulation in vegetative and reproductive parts. The primary objective of this study was to determine the influence of NPK rates and cultivars on the N, P, and K content in dry matter; quantify nutrient partitioning dynamics in maize during the entire growing season; and grain and stover yields at harvest. The second objective was to evaluate macronutrient balances in maize plants, both at total and component levels.

## Materials and Methods

### Site Description

Field trials were set up at a farmer’s field in Gayeshpur, Nadia, West Bengal, India (23°26.01’ N latitude, 88°22.22’ E longitude, 12.0 meters above mean sea level) during two consecutive winter seasons of 2012–13 (year 1) and 2013–14 (year 2). The climate of the region is humid-tropical, with a hot summer and a moderately cold winter. Maximum and minimum temperatures fluctuated between 21.2 and 39.8°C and 6.9 and 25.2°C, respectively, during year 1 and between 23.1 and 41.3°C and 9.2 and 26.6°C, respectively, during year 2. In general, there was a gradual drop in temperature from November to January that favored the growth and development of maize hybrids. Maximum and minimum relative humidity prevailed between 83 and 98% and 25 and 68%, respectively, during year 1 and between 80 and 95% and 35 and 62%, respectively, during year 2. The rainfall during the experimental period (November to March) was 91.4 mm in year 1 and 54.7 mm in year 2. Soil texture of the experimental field was clay-loam. The experiment was conducted under irrigated upland conditions with good drainage, neutral soil pH (7.31), low available N (215.2 kg ha^–1^), high available P (41.6 kg ha^–1^), and medium available K (186.4 kg ha^–1^). The initial physico-chemical properties of the experimental soil (sampled up to 30 cm depth) are summarized in a supplementary table (**S1 File**, Table A).

### Experimental Design and Treatment Details

The study was arranged as a strip plot design with three hybrid maize cultivars (P3522, P3396, and Rajkumar) in the vertical strip, and nine levels of NPK: 50% RDF (N_100_P_30_K_30_); 75% RDF (N_150_P_45_K_45_); 100% RDF (N_200_P_60_K_60_); 125% RDF (N_250_P_75_K_75_); 150% RDF (N_300_P_90_K_90_); 100% P and K (P_60_K_60_, N omission); 100% N and K (N_200_K_60_, P omission); 100% N and P (N_200_P_60_,K omission); and control (zero-NPK)] in the horizontal strip, replicated three times. The 100% RDF plot was considered as the optimum nutrient treatment plot. The selected cultivars had been historically high yielding (Ray et al., 2018; Ray et al., 2019) and were popular with farmers in the study area. There was no specific RDF for hybrid maize for that location before we started the experiment. The recommendation from the Indian Council of Agricultural Research (ICAR)-Indian Institute of Maize Research (IIMR) was used (http://www.iimr.res.in/) to define the RDF as 200 kg N, 60 kg P_2_O_5_, and 60 kg K_2_O ha^–1^. Seeds were sown at 25 kg ha^–1^ on 20th November in both years at a spacing of 60 × 30 cm (55,555 plants ha^–1^). Seeds were dibbled at a 3–5 cm depth, with two seeds at each position. The net plot size was 4 × 3 m, with nine crop rows in each plot. Each plot was separated by a 0.3 m bund. Forty percent of N (as urea) with full doses of P_2_O_5_ (as single super phosphate) and K_2_O (as muriate of potash) was surface broadcasted during final land preparation (as basal). The remainder of the N was top-dressed in two equal splits (30% of N each), at 45 (V6 stage) and 80 (pre-tasseling stage) days after seeding (DAS). The crop was irrigated seven times during both years of the study. The first two irrigations were given respectively at 3 (in year 1) to 5 (in year 2) DAS, and at 35 (in year 1) to 40 (in year 2) DAS (when maize was V6 stage). The next three irrigations were applied from 60 DAS (pre-flowering) onwards at 10–15 days interval. The sixth and seventh irrigations were given at 100 (silking) and 110 (early grain-filling) DAS. The experimental area was hand weeded (twice at 40 and 60 DAS) to control the most dominant weed species, *Chenopodium album* L. Besides hand weeding, a pre-emergence treatment of Metribuzin 480 Suspension Concentrate or SC (Brand name: Sencor) at 1 kg a.i. ha^–1^ in 600 L of water was applied. Only in the first year, Lambda Cyhalothrin 5 Emulsifiable concentrate or EC (Brand name: Agent plus) at 10 ml 15 L^–1^ water was applied (65 DAS) to control grasshopper (*Hieroglyphus* sp.). No insect control was required in the second year.

All selected cultivars used for this study are recent (released after 2010), “stay-green,” and suitable for alluvial soils of West Bengal in a humid tropical region (Ray, 2016). The important characteristics of cultivars P3522 and P3396 include the larger cobs with good tip filling, preferred grain color, soft endosperm type, and good yields under a wide range of environments. Seeds were verified to have 90% germination, 98% physical purity, 95% genetic purity, and were treated with Deltamethrin 2.5% Wettable Powder (WP). The cultivars P3522 and P3396 were produced and marketed by Pioneer (PHI Seeds private Ltd.), Andhra Pradesh, India. The difference between P3396 and P3522 is that the former has more tolerance to stalk rot disease and has uniform grain filling in the cobs (Ray, 2016). The third cultivar ‘Rajkumar,’ produced by Shriram Bioseed Genetics, Hyderabad, Andhra Pradesh, India, is suitable for both winter and rainy seasons in the study region. It has a medium duration growth period and greater grain weight and protein content. The seed color of this cultivar is lustrous orange-yellow, indicating an enrichment of vitamin A (Nuss et al., 2012). The seeds are 98% pure with only 2% inert matter, 10 kg^–1^ other crop seed, and negligible amount of weed seed.

### Measurements for Dry Matter Accumulation, Partitioning, and Remobilization

Three randomly selected plants from destructive sampling (1 m long row) in each plot were cut at ground level at each physiological stage. Plants at both sampling dates, i.e. at 100 (silking or R1 stage) and at 120 (physiological maturity or R6 stage) DAS, were separated into leaves, stems, and reproductive parts (tassel at R1 stage, and tassel, husk, shank, and grain at R6 stage) for analysis. The level of resolution in this study was the plant organ. All leaves were measured as a group rather than as each individual leaf or groups of leaves. Differences among leaves of different ages have been documented (Ning et al., 2013), but this study evaluated the net contribution of all leaves. Plant samples were sun-dried and then transferred to a thermostatically controlled dry oven, regulated at a temperature of 70°C for approximately 24 h until dry weight was constant. Post-silking dry matter accumulation in aboveground biomass (PSDMA), dry matter remobilization efficiency (DMRE) of vegetative tissue, and contribution to grain dry matter by leaf and stalk dry matter remobilization (DMR) were estimated according to the following formulas (Mi et al., 2003; Chen et al., 2014; Chen et al., 2015):

 (1) $${\text{PSDMA (kg ha}}^{-1}\text{)=Total DMA at R}6-\text{Total DMA at R}1$$

(2) $$\begin{array}{l} \text{DMRE }(\%)\text{ of leaf or stalk =}\\ 100\times [\{(\text{leaf or stalk DMA at R}1)-(\text{leaf or stalk DMA at R}6)\}/(\text{leaf or stalk DMA at R}1)] \end{array}$$

(3) $$\begin{array}{l} \text{DMR }(\%)\text{ of leaf or stalk =}\\ 100\times [\{(\text{leaf or stalk DMA at R}1)-(\text{leaf or stalk DMA at R}6)\}/(\text{grain DMA at R}6)] \end{array}$$

Dry matter harvest index or HI(DM) was calculated as per Ciampitti et al. (2013):

 (4) $$\text{HI(DM)=}\frac{\text{Grain DMA at R}6}{\text{Total DMA at R}6}$$

### Measurements for Nutrient Accumulation, Partitioning, and Remobilization

Plant samples (leaf, stem, and reproductive organs), after being measured for dry matter accumulation (as mentioned in *Measurements for Dry Matter Accumulation, Partitioning, and Remobilization* sub-section), were chopped and ground using a stainless steel mechanical grinder before N/P/K concentration (%) analysis. For N analysis, 0.5 g of each plant sample was digested with concentrated H_2_SO_4_ for 1–2 h at 420°C until green color was obtained. The digest was distilled with 40% NaOH and back-titrated with 0.025 (N) H_2_SO_4_ by Micro-Kjeldahl’s method (AOAC, 1995). For the estimation of P and K from plant samples, 1 g of the ground sample was placed in a 100 ml glass conical flask and 15 ml of mixed triple acid mixture (HNO_3_: H_2_SO_4_: HClO_4_:: 9:1:4) was added, and digested till the acid liquid had been completely volatilized and a colorless solution obtained as described by Jackson (1973). The digest was diluted, filtered through Whatman No. 42 filter paper and the filtrate was diluted to a volume of 50 ml with distilled water. The aliquot was used for determination of P and K using a UV-VIS spectrophotometer (P) and flame photometer (K) following Jackson (1973). Accumulation of nutrients (N/P/K) in each plant part was calculated by multiplying the nutrient concentration (%) by the dry matter accumulation of each plant part, divided by 100, and expressed as kg ha^–1^. Nutrient partitioning and remobilization were estimated based on the data of plant nutrient accumulation by using the following parameters as suggested by some investigators (Mi et al., 2003; Chen et al., 2014; Chen et al., 2015). Post-silking N, P, or K accumulations (PSNA, PSPA, PSKA), derived from N, P, and K accumulations at R1 and R6:

$$\text{PSNA}(\text{kg N h}a^{-1})=\text{Total NA at R}6-\text{Total NA at R}1$$

$$\text{PSPA}(\text{kg P h}a^{-1})=\text{Total PA at R}6-\text{Total PA at R}1$$

(5) $$\text{PSKA}(\text{kg K h}a^{-1})=\text{Total KA at R}6-\text{Total KA at R}1$$

Remobilization efficiencies of N, P, or K (NRE, PRE, KRE) of leaf or stalk:

$$\begin{array}{l} \text{NRE }(\%)\\ =100\times [\{(\text{leaf or stalk NA at R}1)-(\text{leaf or stalk NA at R}6)\}/(\text{leaf or stalk NA at R}1)] \end{array}$$

$$\begin{array}{l} \text{PRE }(\%)\\ =100\times [\{(\text{leaf or stalk PA at R}1)-(\text{leaf or stalk PA at R}6)\}/(\text{leaf or stalk PA at R}1)] \end{array}$$

(6) $$\begin{array}{l} \text{KRE }(\%)\\ =100\times [\{(\text{leaf or stalk KA at R}1)-(\text{leaf or stalk KA at R}6)\}/(\text{leaf or stalk KA at R}1)] \end{array}$$

Contribution to grain N, P, and K accumulation by remobilization (NR, PR, KR) from leaf or stalk:

NR (%)=100×[{(leaf or stalk NA at R1)−(leaf or stalk NA at R6)}/grain NA at R6]

PR (%)=100×[{(leaf or stalk PA at R1)−(leaf or stalk PA at R6)}/grain PA at R6]

(7) KR (%)=100×[{(leaf or stalk KA at R1)−(leaf or stalk KA at R6)}/grain KA at R6]

Nutrient harvest indices for N, P, and K i.e. HI(N), HI(P), HI(K) were calculated according to Ciampitti et al. (2013):

$$\text{HI(N)=}\frac{\text{grain NA at R}6}{\text{total NA at R}6}$$

$$\text{HI(P)=}\frac{\text{grain PA at R}6}{\text{total PA at R}6}$$

(8) $$\text{HI(K)=}\frac{\text{grain KA at R}6}{\text{total KA at R}6}$$

### Grain and Stover Yield

Plants from the demarcated net plot area (12 m^2^) were harvested and tied in bundles after removing all the matured cobs from them. Grains were shelled from the cob (dehusked) and then dried properly to reduce the moisture content to 14.0%. Weight of grains was recorded as kg plot^–1^ and then converted into Mg ha^–1^. The plants (without grain) were dried in the sun and finally weighed to obtain stover yield in kg ha^–1^ and then converted into Mg ha^–1^.

### Statistical Analysis

Data were subjected to analysis of variance (ANOVA) appropriate for a strip plot design and the mean values were separated by Tukey’s HSD (honest significant difference) test using GenStat (20th Edition, VSN International, Hemel Hempstead, UK, web page: Genstat.co.uk) software. The variance over years was estimated to be homogeneous by performing Bartlett’s chi-square test, and therefore the year variance was pooled with the experimental error variance.

## Results

### Dry Matter Partitioning at Different Growth Stages

The DMA (g plant^–1^) in different plant parts at silking and physiological maturity stages, and PSDMA (g plant^–1^) as influenced by year, cultivar, and levels of NPK are described in **Table 1**. The study highlights that the cultivars as well as the NPK applications significantly (*p* ≤ 0.05) influenced DMA in different plant parts (leaf, stem, and tassel) of maize at silking and at the physiological maturity stages. However, the year effect was non-significant (*p* ≥ 0.05) on DMA in maize plant parts at similar physiological stages (**Table 1**).

**Table 1:** Effects of year, cultivar, and levels of NPK on dry matter accumulation (DMA, g plant^–1^) in different plant organs at different growth stages and on dry matter harvest index of maize.

Treatments	At silking				At physiological maturity					Post-silking DM accumulation	HI (DM)^†^
	Leaf	Stalk		Total	Leaf	Stalk		Grain	Total		
		Stem	Tassel			Stem	Other				
*Year*											
Year 1	39.2a	129.5a	5.3a	174.1a	49.0a	121.1a	52.3a	182.5a	405.0a	230.8a	0.45a
Year 2	40.0a	126.6a	5.4a	171.1a	52.2a	119.2a	52.5a	181.2a	405.4a	234.3a	0.45a
*Cultivar*											
P3522	44.3a	140.1a	2.8c	187.1a	53.7a	135.5a	50.9b	188.6a	428.7a	241.6a	0.44a
P3396	34.3c	121.1b	5.0b	160.4b	49.0b	116.0b	56.5a	180.0b	401.4b	241.0a	0.45a
Rajkumar	39.0b	123.0b	8.3a	170.4b	49.6b	108.9b	49.8b	177.1b	385.4b	215.0b	0.46a
*Levels of NPK*											
N_100_P_30_K_30_ (50% RDF)	39.0b	118.7d	5.2c	162.9c	43.4c	107.9cd	51.4c	168.9d	371.6c	208.7c	0.46a
N_150_P_45_K_45_ (75% RDF)	42.1ab	130.0cd	5.5bc	177.7bc	51.7b	120.3bc	53.3c	197.2bc	422.6b	242.4b	0.47a
N_200_P_60_K_60_ (100% RDF)	44.3a	151.1b	5.9ab	201.4a	60.5a	142.6b	58.3b	209.7b	471.1a	269.7ab	0.45a
N_250_P_75_K_75_ (125% RDF)	44.3a	157.8a	6.2a	208.3a	61.4a	152.8a	62.3a	211.6a	488.1a	279.8a	0.43a
N_300_P_90_K_90_ (150% RDF)	43.8a	156.1a	6.2a	206.1a	55.4ab	135.6b	58.1b	224.5a	473.5a	267.4ab	0.48a
P_60_K_60_ (100% RDF–N)	30.0c	88.7e	4.3d	123.0d	41.5c	94.8de	42.0d	120.8e	299.1d	176.1d	0.41a
N_200_K_60_ (100% RDF–P)	41.8ab	141.4bc	6.0a	189.3ab	51.7b	127.5abc	53.7c	199.4bc	432.3b	243.6b	0.46a
N_200_P_60_ (100% RDF–K)	39.8b	128.9cd	5.0c	173.8bc	53.6ab	120.6bc	52.9c	189.2c	416.3b	244.5b	0.46a
Control (–NPK)	27.5c	79.7e	4.1d	111.3d	37.5c	79.3e	39.7d	115.5e	272.0d	160.8d	0.43a
*Source of variation*											
Year	ns	ns	ns	ns	ns	ns	ns	ns	ns	ns	ns
Cultivar	**	*	**	**	*	*	**	*	*	*	ns
Levels of NPK	**	**	**	**	**	**	**	**	**	**	ns
Year × cultivar	ns	ns	ns	ns	ns	ns	ns	ns	ns	ns	ns
Year × levels of NPK	ns	ns	ns	ns	ns	ns	ns	ns	ns	ns	ns
Cultivar × levels of NPK	*	**	ns	**	ns	ns	*	*	ns	*	ns
Year × cultivar × levels of NPK	ns	ns	ns	ns	ns	ns	ns	ns	ns	ns	ns

Year 1 and year 2 represent winter 2012–13 and 2013–14, respectively. Within year, cultivar, and levels of NPK, numbers followed by different letters indicate significant differences at p ≤ 0.05 (otherwise statistically at par). ns, non-significant (p > 0.05). *Significant at p ≤ 0.05. **Significant at p ≤ 0.01; Other = Tassel + cob + shank + husk; ^†^HI (DM), Harvest Index (Dry Matter)

At silking, the cultivar P3522 exhibited the highest leaf and stem dry matter accumulation followed by Rajkumar and P3396. The cultivar Rajkumar, however, produced the highest DMA in the tassel, while P3522 exhibited lowest tassel dry matter. The total DMA followed exactly the same trend to that of leaf dry matter. Overall, the DMA was distributed more in the stem than in the leaves across all NPK treatments. At silking, applied NPK up to 100% RDF significantly (*p* ≤ 0.05) increased leaf dry matter (**Table 1**). Stem and tassel dry matter significantly (*p* ≤ 0.05) increased with successive increase in NPK levels up to 125% RDF. Although the numerically highest total DMA was obtained with 125% NPK fertilization, it was statistically at par with 100 and 150% NPK fertilization. Omission of N and K caused significant (*p* ≤ 0.05) reductions in DMA in different plant parts as well as total DMA. When compared with 100% RDF, the reduction in DMA was lowest with P omission (100% NK) and highest with N omission (100% PK). Control (–NPK) treatment recorded lowest total DMA as well as leaf, stem, and tassel dry matter.

At physiological maturity, cultivars showed significant (*p* ≤ 0.05) differences with respect to DMA in different plant parts as well as total DMA. Leaf, stem, and grain dry matter was significantly (*p* ≤ 0.05) higher in P3522, but highest DMA in other parts of the stalk (tassel + cob + shank + husk) was recorded in P3396. Overall, across different NPK treatments, highest DMA was observed in grain followed by stem, other parts of the stalk (tassel + cob + shank + husk) and leaves at physiological maturity. An NPK rate of 100% RDF maximized leaf DMA, but a higher rate (125% RDF) was needed to maximize stalk DMA (both stem and other) as well as grain DMA. Further increases in NPK levels up to 150% RDF did not produce any additional gains in leaf or grain DMA but did reduce stalk DMA. When omitting individual nutrients, reduction in DMA (total as well as different plant parts) at physiological maturity was greatest with N omission, followed by K and P omission. When compared with 100% RDF, total dry matter reduction with N, P, and K omission, respectively, was 38.9, 6.0, and 13.7% at silking, and 36.5, 8.2, and 11.6% at physiological maturity. The control (–NPK) treatment caused the greatest reduction in total DMA as well as leaf, stem, and tassel DMA.

The variation in PSDMA was non-significant over the study years (**Table 1**). Cultivars P3522 and P3396 had the highest PSDMA and were both significantly (*p* ≥ 0.05) greater than that of Rajkumar. An NPK rate of 125% RDF maximized PSDMA. Omission of N caused a significant reduction in PSDMA; however, the lowest PSDMA was recorded in control (–NPK) plots. Numerical reductions in PSDMA also occurred with P and K omission but were non-significant (*p* ≥ 0.05).

Dry matter harvest index [HI(DM)] did not show any significant variation due to year, cultivar, or levels of NPK. The stability of HI(DM) indicates that the three maize cultivars used in this study maintained a constant proportion of their accumulated dry matter in the grain regardless of the imposed conditions. The average HI(DM) was 0.45.

### Dry Matter Remobilization and Its Contribution to Grain Dry Matter Accumulation

The dry matter accumulated in vegetative tissues can be remobilized and can contribute to crop yield. Remobilization describes movement of dry matter and nutrients from their initial location of accumulation (leaf, stalk) to other tissues, typically the grain. The DMRE (%) and DMR (%) as influenced by year, cultivar, and levels of NPK are described in **Table 2**. All of the DMRE and DMR values were negative, meaning that the net behavior of the leaf and stalk tissue was that of a sink rather than a source for dry matter remobilization.

**Table 2:** Dry matter remobilization efficiency (%) and contribution to grain dry matter (%) in maize as influenced by year, cultivar, and levels of NPK.

Treatments	DM remobilization efficiency		Contribution to grain DM by DM remobilization	
	Leaf	Stalk	Leaf	Stalk
*Year*				
Year 1	−27.8b	−32.0a	−5.57b	−21.6a
Year 2	−37.1a	−37.0a	−7.86a	−23.7a
*Cultivar*				
P3522	−23.7b	−35.8b	−5.56a	−25.6ab
P3396	−45.4a	−44.1a	−8.40a	−27.7a
Rajkumar	−28.2b	−23.6b	−6.17a	−14.6b
*Levels of NPK*				
N_100_P_30_K_30_ (50% RDF)	−15.1d	−33.2b	−3.07b	−20.8ab
N_150_P_45_K_45_ (75% RDF)	−23.1c	−29.3bc	−5.10ab	−18.9ab
N_200_P_60_K_60_ (100% RDF)	−37.5ab	−34.8b	−7.77ab	−21.2ab
N_250_P_75_K_75_ (125% RDF)	−39.7a	−32.7b	−8.31ab	−24.7ab
N_300_P_90_K_90_ (150% RDF)	−31.0ab	−19.5c	−5.19ab	−13.9b
P_60_K_60_ (100% RDF–N)	−41.6a	−55.8a	−9.23a	−35.3a
N_200_K_60_ (100% RDF–P)	−28.1bc	−23.6bc	−4.80ab	−17.1ab
N_200_P_60_ (100% RDF–K)	−35.9ab	−30.7b	−7.76ab	−21.5ab
Control (–NPK)	−40.1a	−51.2a	−9.20a	−30.5ab
*Source of variation*				
Year	*	ns	ns	ns
Cultivar	*	*	ns	*
Levels of NPK	*	*	*	*
Year × cultivar	ns	ns	*	ns
Year × levels of NPK	ns	ns	ns	ns
Cultivar × levels of NPK	ns	*	ns	*
Year × cultivar × levels of NPK	ns	ns	ns	ns

Year 1 and year 2 represent winter 2012–13 and 2013–14, respectively. Within year, cultivar, and levels of NPK, numbers followed by different letters indicate significant differences at p ≤ 0.05 (otherwise statistically at par). ns, non-significant (p > 0.05). *Significant at p ≤ 0.05. – sign represents the situations when the tissue is acting as a sink.

For DMRE, the effect of year was significant (*p* ≤ 0.05) only for leaf dry matter, with year 2 having lower (more negative) DMRE. Cultivar P3396 exhibited significantly (*p* ≤ 0.05) lower leaf and stalk DMRE, than P3522 and Rajkumar, both of which were similar. The varietal difference in DMR exhibited almost the same trend as was observed in case of DMRE. Levels of NPK exerted significant (*p* ≤ 0.05) influence on DMRE and DMR (**Table 2**). With subsequent increase in NPK levels up to 150% RDF, dry matter remobilization and its contribution to grain dry matter were decreased for leaves and stalks. It is interesting to note that nutrient omission and control (–NPK) treatments caused poor vegetative dry matter remobilization and contributed less to grain dry matter. Both leaf and stalk dry matter remobilization and their subsequent contribution to grain dry matter were observed to be the lowest in case of N-omission plots and were statistically at par with absolute control plots.

### Nitrogen Partitioning at Different Growth Stages

Nitrogen accumulation in different plant parts and post-silking N accumulation as influenced by year, cultivar, and levels of NPK are described in **Table 3**.

**Table 3:** Effects of year, cultivar, and levels of NPK on nitrogen accumulation (kg ha^–1^) in different plant organs at different growth stages and on nitrogen harvest index of maize.

Treatments	At silking				At physiological maturity					Post-silking N accumulation	HI(N)^†^
	Leaf	Stalk		Total	Leaf	Stalk		Grain	Total		
		Stem	Tassel			Stem	Other				
*Year*											
Year 1	51.5a	55.6a	7.66a	114.8a	44.2b	15.6b	9.21a	114.8a	183.8b	69.0b	0.64a
Year 2	51.6a	54.8b	7.68a	114.1a	52.4a	22.0a	9.20a	118.2a	201.8a	87.7a	0.58b
*Cultivar*											
P3522	63.3a	59.4a	3.9c	126.7a	49.6a	14.7c	7.2b	122.2a	193.7a	67.0b	0.63a
P3396	42.9c	54.6b	7.4b	105.0c	47.7a	18.7b	10.3a	118.6a	195.4a	90.4a	0.61a
Rajkumar	48.3b	51.7b	11.7a	111.7b	47.6a	23.0a	10.1a	108.7b	189.4a	77.8ab	0.58a
*Levels of NPK*											
N_100_P_30_K_30_ (50% RDF)	49.6e	42.2e	6.8ef	98.5d	50.0cd	15.8de	8.0d	118.1cd	191.8e	93.3bc	0.61ab
N_150_P_45_K_45_ (75% RDF)	58.1cd	55.7d	8.0cd	121.9c	53.7bc	18.7d	10.1c	129.3bc	211.9d	90.0bc	0.61ab
N_200_P_60_K_60_ (100% RDF)	64.8ab	71.6c	8.9bc	145.2b	60.0b	23.2c	11.6b	144.3ab	239.1c	93.9bc	0.60ab
N_250_P_75_K_75_ (125% RDF)	68.8a	80.1b	9.3a	158.2a	67.9a	29.8b	12.0b	149.6a	259.2b	101.0ab	0.58ab
N_300_P_90_K_90_ (150% RDF)	60.7bc	90.3a	9.6a	160.6a	70.3a	41.7a	14.0a	153.3a	279.2a	118.6a	0.55b
P_60_K_60_ (100% RDF–N)	28.2f	25.0f	5.9fg	59.1e	29.5e	8.7gh	6.3e	62.7e	107.2g	48.2d	0.59ab
N_200_K_60_ (100% RDF–P)	57.7cd	56.0d	8.3bc	122.1c	45.7d	13.7ef	8.3d	129.8bc	197.4de	75.4c	0.66a
N_200_P_60_ (100% RDF–K)	54.1de	57.6d	7.1de	118.7c	36.4e	10.7fg	7.5d	107.4d	161.9f	43.2d	0.66a
Control (–NPK)	21.7g	18.8g	5.2g	45.7f	21.2f	7.0h	5.1f	54.1e	87.5h	41.8d	0.62ab
*Source of variation*											
Year	ns	*	ns	ns	*	**	ns	ns	*	**	**
Cultivar	**	*	**	**	ns	**	**	*	ns	*	ns
Levels of NPK	**	**	**	**	**	**	**	**	**	**	**
Year × cultivar	ns	*	ns	ns	*	*	ns	ns	ns	ns	*
Year × levels of NPK	ns	*	ns	ns	ns	*	ns	ns	ns	ns	ns
Cultivar × levels of NPK	**	**	ns	**	ns	**	**	ns	ns	ns	ns
Year × cultivar × levels of NPK	ns	*	ns	ns	ns	ns	ns	ns	ns	ns	ns

Year 1 and year 2 represent winter 2012–13 and 2013–14, respectively. Within year, cultivar, and levels of NPK, numbers followed by different letters indicate significant differences at p ≤ 0.05 (otherwise statistically at par). ns, non-significant (p > 0.05). *Significant at p ≤ 0.05. **Significant at p ≤ 0.01; Other = Tassel + cob + shank + husk; ^†^HI (N), Harvest Index (Nitrogen)

The year effect was significant (*p* ≤ 0.05) for stem N accumulation at silking and post-silking stage, and for leaf and stem N accumulation at physiological maturity stage, resulting in significant (*p* ≤ 0.05) variation in total N accumulation (**Table 3**). The year effect was significant for post-silking N accumulation (PSNA), and it was higher in year 2 than year 1.

At silking, partitioning of N in different plant parts varied significantly (*p* ≤ 0.05) among cultivars. Cultivar P3522 produced the highest (*p* ≤ 0.05) N accumulation in leaf and stem, ultimately resulting in greater total N accumulation. However, the cultivar Rajkumar accumulated more N in the tassel. At physiological maturity, leaf N accumulation exhibited non-significant (*p* ≥ 0.05) variation in tested cultivars. The cultivar Rajkumar accumulated more N in stalk while other parts of the stalk accumulated higher N in P3396. Cultivar P3522 and P3396 had no significant difference regarding grain N accumulation; however, both the cultivars accumulated significantly (*p* ≤ 0.05) more grain N than cultivar Rajkumar. There was non-significant (*p* > 0.05) variation in total N accumulation among the tested cultivars. At post-silking, P3396 accumulated significantly (*p* ≤ 0.05) more N than P3522.

Both at silking and physiological maturity stages, successive increases in levels of NPK up to 150% RDF augmented N accumulation in stalk, total plant part and grain, ultimately resulting in higher PSNA. Reduction of N accumulation in all plant parts was greater with N omission over P and K omission. When compared with 100% RDF, reduction in total N accumulation was 59.3, 15.9, and 18.3% at silking, and 55.2, 17.4, and 32.3% at physiological maturity due to N, K, and P omission, respectively. Moreover, control (–NPK) treatments resulted in drastic reductions in N accumulation in all plant parts.

Nitrogen harvest index or HI(N) was significantly (*p* ≤ 0.05) higher in the first year than the second year, and it remained unaffected by cultivar. Successive increases in NPK levels decreased HI(N), with the lowest value obtained with 150% RDF. Among different nutrient management treatments, omission plots showed comparatively higher HI(N); whereas P and K omission treatments produced highest (*p* ≤ 0.05) HI(N).

### Nitrogen Remobilization and Its Contribution to Grain Nitrogen Accumulation

Nitrogen remobilization efficiency (%) and contribution to grain nitrogen (%) as influenced by year, cultivar, and levels of NPK are described in **Table 4**.

**Table 4:** Nitrogen remobilization efficiency (%) and contribution to grain nitrogen (%) in maize as influenced by year, cultivar, and levels of NPK.

Treatments	N remobilization efficiency		Contribution to grain N by N remobilization	
	Leaf	Stalk	Leaf	Stalk
*Year*				
Year 1	+10.5a	+60.3a	7.6a	33.7a
Year 2	−11.9b	+47.7b	−2.1b	26.4b
*Cultivar*				
P3522	+20.5a	+64.4a	11.1a	33.8a
P3396	−17.0b	+50.2b	−5.0c	27.1b
Rajkumar	−5.5b	+47.4b	2.1b	29.2b
*Levels of NPK*				
N_100_P_30_K_30_ (50% RDF)	−17.7de	+51.0cd	−1.4cd	22.0d
N_150_P_45_K_45_ (75% RDF)	−0.1bcd	+53.7c	3.1bc	27.0bcd
N_200_P_60_K_60_ (100% RDF)	+5.4bc	+56.2bc	2.7bc	32.9b
N_250_P_75_K_75_ (125% RDF)	+0.5bcd	+52.5cd	0.4cd	32.4b
N_300_P_90_K_90_ (150% RDF)	−20.3e	+43.8d	−7.1d	30.1bc
P_60_K_60_ (100% RDF–N)	−16.1de	+48.0cd	−3.1cd	24.3cd
N_200_K_60_ (100% RDF–P)	+12.0bc	+63.1bc	10.0a	33.7b
N_200_P_60_ (100% RDF–K)	+30.9a	+70.6a	18.2a	45.5a
Control (–NPK)	−0.6bcd	+47.2cd	1.7bcd	22.3d
*Source of variation*				
Year	*	**	*	**
Cultivar	**	**	*	*
Levels of NPK	**	**	**	**
Year × cultivar	ns	ns	ns	ns
Year × levels of NPK	ns	ns	*	ns
Cultivar × levels of NPK	**	**	**	*
Year × cultivar × levels of NPK	ns	ns	ns	ns

Year 1 and year 2 represent winter 2012–13 and 2013–14, respectively. Within year, cultivar, and levels of NPK, numbers followed by different letters indicate significant differences at p ≤ 0.05 (otherwise statistically at par). ns, non-significant (p > 0.05). *Significant at p ≤ 0.05. **Significant at p ≤ 0.01, + and − sign represent the situations when the tissue is acting as a source and sink, respectively.

The N remobilization efficiency and its contribution to grain N was significantly (*p* ≤ 0.05) higher in year 1 than year 2 (**Table 4**). The highest vegetative N remobilization and its contribution to grain N was observed in P3522 irrespective of the plant parts studied; however, that same cultivar exhibited the lowest PSNA (**Table 3**). The stalk N remobilization and its contribution to grain N was comparatively higher than that of leaves. The maximum reduction in PSNA was observed with K omission, followed by N and P omission, respectively (**Table 3**); however, leaf and stalk N remobilization efficiency and their contribution to grain N were significantly greater with K omission, followed by P and N omission (**Table 4**).

### Phosphorus Partitioning at Different Growth Stages

The year effect was non-significant (*p* > 0.05) for P accumulation in different plant parts as well as in the total plant at silking and physiological maturity stages (**Table 5**). At the silking stage, significant (*p* ≤ 0.05) differences were found among maize cultivars for P accumulation in various plant parts. Greater P accumulation in leaves were observed in cultivars P3522 and Rajkumar. Significantly higher stem and tassel P accumulations were recorded in P3522 and P3396, respectively. The total P accumulation was higher in P3522, which was similar to the total P accumulation in Rajkumar. Phosphorus accumulations in stem, tassel, and in the total plant at silking were significantly increased by increasing levels of NPK up to 150% RDF. At physiological maturity the cultivar P3396 accumulated significantly higher P in leaf and stalk, resulting in higher P accumulation in the whole plant (**Table 5**); however, P3522 exhibited the highest P accumulation in the grain. The lowest grain P accumulation was observed in Rajkumar and was not significantly different than that of P3396. Among the tested cultivars, P3396 had the highest PSPA and consequently the lowest P remobilization and its contribution to grain P (**Table 6**).

**Table 5:** Effects of year, cultivar, and levels of NPK on phosphorus accumulation (kg ha^–1^) in different plant organs at different growth stages and on phosphorus harvest index of maize.

Treatments	At silking				At physiological maturity					Post-silking P accumulation	HI(P)^†^
	Leaf	Stalk		Total	Leaf	Stalk		Grain	Total		
		Stem	Tassel			Stem	Other				
*Year*											
Year 1	7.1a	15.0a	1.4a	23.4a	4.9a	8.1a	2.7a	30.6a	46.3a	23.0a	0.67a
Year 2	7.2a	14.4a	1.5a	23.1a	5.0a	7.0a	2.5a	28.9a	43.4a	20.4b	0.68a
*Cultivar*											
P3522	8.0a	15.9a	0.7c	24.5a	4.8b	5.5b	2.1c	32.6a	45.0a	20.4b	0.74a
P3396	5.7b	13.7b	2.2a	20.8b	5.7a	9.1a	3.4a	28.9b	47.1a	26.3a	0.62b
Rajkumar	7.8a	14.3b	1.4b	24.3a	4.5b	8.1a	2.4b	27.7b	42.6b	18.3b	0.66b
*Levels of NPK*											
N_100_P_30_K_30_ (50% RDF)	6.4d	15.1bc	1.4cd	22.9d	5.1cde	6.8cde	2.1d	28.9ef	42.9d	20.0cd	0.67ab
N_150_P_45_K_45_ (75% RDF)	7.0cd	15.7bc	1.5bc	24.2cd	5.6bcd	7.7cd	2.6c	33.0cd	48.9c	24.7bc	0.68ab
N_200_P_60_K_60_ (100% RDF)	8.3b	16.3b	1.6abc	26.1bc	6.1bc	9.0bc	3.2b	35.5bc	53.7b	27.6b	0.66ab
N_250_P_75_K_75_ (125% RDF)	8.9a	16.9b	1.7ab	27.5b	7.5a	16.4a	4.0a	38.8a	66.7a	39.3a	0.59b
N_300_P_90_K_90_ (150% RDF)	9.2a	19.4a	1.8a	30.4a	6.5b	11.0b	2.8c	36.9ab	57.3b	26.8b	0.65ab
P_60_K_60_ (100% RDF–N)	5.4e	11.7e	1.1e	18.2f	3.2fg	4.2ef	1.9de	20.1g	29.4f	11.2ef	0.69a
N_200_K_60_ (100% RDF–P)	6.8cd	12.6de	1.3d	20.7e	3.8efg	4.9def	2.6c	26.2f	37.6e	16.8de	0.70a
N_200_P_60_ (100% RDF–K)	7.4c	14.3cd	1.4cd	23.1d	4.4def	5.9de	2.6c	30.5de	43.3d	20.2cd	0.71a
Control (–NPK)	5.0e	9.7f	1.1e	15.9g	2.7g	2.3f	1.7e	17.6g	24.2g	8.4f	0.73a
*Source of variation*											
Year	ns	ns	ns	ns	ns	ns	ns	ns	ns	*	ns
Cultivar	**	*	**	**	*	*	**	**	*	**	*
Levels of NPK	**	**	**	**	**	**	**	**	**	**	**
Year × cultivar	ns	ns	ns	ns	ns	ns	ns	*	ns	ns	ns
Year × levels of NPK	ns	ns	ns	ns	ns	ns	ns	ns	ns	ns	ns
Cultivar × levels of NPK	**	ns	ns	**	ns	ns	**	*	ns	*	ns
Year × cultivar × levels of NPK	ns	ns	ns	ns	ns	ns	ns	ns	ns	ns	ns

Year 1 and year 2 represent winter 2012–13 and 2013–14, respectively. Within year, cultivar, and levels of NPK, numbers followed by different letters indicate significant differences at p ≤ 0.05 (otherwise statistically at par). ns, non-significant (p > 0.05). *Significant at p ≤ 0.05. **Significant at p ≤ 0.01; Other = Tassel + cob + shank + husk; ^†^HI(P), Harvest Index (Phosphorus)

**Table 6:** Phosphorus remobilization efficiency (%) and contribution to grain P (%) in maize as influenced by year, cultivar, and levels of NPK.

Treatments	P remobilization efficiency		Contribution to grain P by P remobilization	
	Leaf	Stalk	Leaf	Stalk
*Year*				
Year 1	+26.4a	+35.6a	7.7a	20.2a
Year 2	+27.7a	+38.8a	8.2a	25.0a
*Cultivar*				
P3522	+41.0a	+56.6a	10.6a	30.0a
P3396	−1.9b	+18.5c	0.9b	12.9b
Rajkumar	+42.0a	+36.6b	12.4a	24.9a
*Levels of NPK*				
N_100_P_30_K_30_ (50% RDF)	+8.0c	+44.7abc	4.9cd	27.0bc
N_150_P_45_K_45_ (75% RDF)	+14.6c	+38.1bc	4.1d	22.2bc
N_200_P_60_K_60_ (100% RDF)	+21.3bc	+30.0c	6.1bcd	16.1c
N_250_P_75_K_75_ (125% RDF)	+9.5c	−13.6d	4.0d	–4.8d
N_300_P_90_K_90_ (150% RDF)	+28.8bc	+32.8bc	7.6bcd	19.8c
P_60_K_60_ (100% RDF–N)	+36.3bc	+51.7ab	10.8b	34.1ab
N_200_K_60_ (100% RDF–P)	+41.6a	+45.4abc	10.9b	24.7bc
N_200_P_60_ (100% RDF–K)	+39.0a	+43.1bc	10.2b	24.3bc
Control (–NPK)	+44.2a	+62.8a	13.3a	40.0a
*Source of variation*				
Year	ns	ns	ns	ns
Cultivar	**	**	**	*
Levels of NPK	**	**	**	**
Year × cultivar	ns	ns	ns	ns
Year × levels of NPK	ns	ns	ns	ns
Cultivar × levels of NPK	*	ns	ns	ns
Year × cultivar × levels of NPK	ns	ns	ns	ns

Year 1 and year 2 represent winter 2012–13 and 2013–14, respectively. Within year, cultivar, and levels of NPK, numbers followed by different letters indicate significant differences at p ≤ 0.05 (otherwise statistically at par). ns, non-significant (p > 0.05). *Significant at p ≤ 0.05. **Significant at p ≤ 0.01, + and − sign represent the situations when the tissue is acting as a source and sink, respectively.

The 125% RDF application resulted in highest P (*p* ≤ 0.05) accumulation in leaf, stalk, total plant part, and grain at physiological maturity, and beyond that level NPK fertilization did not bring about any increase in P accumulation. The reduction in P accumulation in various parts and total plant by nutrient omission treatments was in the order –N > –P > –K, both at silking and physiological maturity stages. When compared with 100% RDF, reduction in total P accumulation was 30.3, 20.7, and 11.5% at silking, and 45.3, 30.0, and 19.4% at physiological maturity due to N, P, and K omission, respectively. During both growth stages, the control (–NPK) treatment reduced P accumulation to a large extent. The application of 125% RDF brought about a significant (*p* ≤ 0.05) increase in PSPA over other NPK levels, while the lowest PSPA was recorded in plants receiving the control treatment (–NPK).

The variation in HI(P) was non-significant (*p* ≥ 0.05) over the study years. Both cultivar and NPK factors exerted significant (*p* < 0.05) influence on HI(P). P3522 had higher HI(P) over two other tested cultivars. Nutrient omission (N/P/K or total NPK) plots registered significantly higher HI(P) and were statistically equivalent.

### Phosphorus Remobilization and Its Contribution to Grain Phosphorus Accumulation

The year effect was non-significant (*p* > 0.05) for P remobilization efficiency and its contribution to grain P, while both the indices were significantly influenced by cultivar factor (**Table 6**). Highest leaf and stalk P remobilization were observed in Rajkumar and P3522, respectively. As an obvious consequence, both the cultivars contributed more to grain P by leaf and stalk P remobilization, and were not statistically different.

The levels of NPK-fertilization exerted significant influence on P mobilization efficiency and its contribution to grain P. Both indices exhibited higher values with nutrient omission treatments; however, the absolute control (–NPK) treatment brought about greater leaf and stalk P remobilization efficiencies and contributions to grain P. The P omission treatment caused greater leaf P remobilization efficiency and its contribution to grain P. The N omission treatment produced greater stalk P remobilization efficiency and contribution to grain P.

### Potassium Partitioning at Different Growth Stages

Potassium accumulation (kg K ha^–1^) in different plant parts and post-silking K accumulation (kg K ha^–1^) of maize as influenced by year, cultivar, and levels of NPK are described in **Table 7**.

**Table 7:** Effects of year, cultivar, and levels of NPK on potassium accumulation (kg ha^–1^) in different plant organs at different growth stages and on potassium harvest index of maize.

Treatments	At silking				At physiological maturity					Post-silking K accumulation^$^	HI(K)^†^
	Leaf	Stalk		Total	Leaf	Stalk		Grain	Total		
		Stem	Tassel			Stem	Other				
*Year*											
Year 1	31.7a	119.5a	4.2a	155.3a	27.2a	48.6a	14.6b	40.0a	130.4a	−24.9a	0.32a
Year 2	31.8a	119.9a	4.1a	155.8a	28.2a	46.0a	15.4a	35.0b	124.6a	−31.2a	0.29b
*Cultivar*											
P3522	35.2a	132.4a	2.3c	169.9a	27.6ab	52.4a	11.7b	39.8a	131.6a	−38.3b	0.31a
P3396	28.4c	119.3b	3.5b	151.2b	31.5a	42.8a	16.9a	37.1b	128.3a	−22.9a	0.30a
Rajkumar	31.8b	107.4c	6.5a	145.6b	24.0b	46.6a	16.4a	35.6b	122.6a	−23.0a	0.30a
*Levels of NPK*											
N_100_P_30_K_30_ (50% RDF)	27.5ef	93.7ef	4.3bc	125.5ef	25.6c	45.9cde	14.9cd	37.8b	124.3d	−1.29a	0.31a
N_150_P_45_K_45_ (75% RDF)	31.5cd	126.0c	4.4bc	162.0c	31.2b	50.3bcd	16.3bc	43.2a	141.0c	−21.0ab	0.32a
N_200_P_60_K_60_ (100% RDF)	37.2b	146.7b	4.8ab	188.7b	33.9b	55.0bc	17.3b	43.7a	149.9bc	−38.7bc	0.30a
N_250_P_75_K_75_ (125% RDF)	40.7a	178.4a	5.3a	224.4a	38.8a	69.1a	20.3a	47.2a	175.4a	−49.0c	0.27a
N_300_P_90_K_90_ (150% RDF)	39.8ab	153.4b	4.9ab	198.1b	34.8b	59.7b	19.3a	45.8a	159.5ab	−38.5bc	0.30a
P_60_K_60_ (100% RDF–N)	25.9f	85.8fg	3.4d	115.1fg	20.2d	34.0de	11.4f	25.3d	90.8e	−24.2ab	0.30a
N_200_K_60_ (100% RDF–P)	33.6c	114.9cd	3.8cd	152.3cd	25.4c	43.3cde	13.7de	38.1b	120.4d	−31.8bc	0.32a
N_200_P_60_ (100% RDF–K)	29.5de	102.7de	3.5d	135.7de	23.0c	38.5cde	12.5ef	33.7c	107.7d	−27.9bc	0.32a
Control (–NPK)	20.2g	75.6g	2.6e	98.5g	16.4e	29.7e	9.4g	22.9d	78.4e	−20.1ab	0.30a
*Source of variation*											
Year	ns	ns	ns	ns	ns	ns	**	**	ns	ns	*
Cultivar	**	**	**	**	*	ns	**	**	ns	*	ns
Levels of NPK	**	**	**	**	**	**	**	**	**	*	ns
Year × cultivar	ns	ns	ns	ns	ns	ns	*	ns	ns	ns	ns
Year × levels of NPK	*	**	ns	**	ns	ns	ns	ns	ns	ns	ns
Cultivar × levels of NPK	ns	ns	ns	ns	ns	ns	ns	ns	ns	ns	ns
Year × cultivar × levels of NPK	ns	ns	ns	ns	ns	ns	ns	ns	ns	ns	ns

Year 1 and year 2 represent winter 2012–13 and 2013–14, respectively. Within year, cultivar, and levels of NPK, numbers followed by different letters indicate significant differences at p ≤ 0.05 (otherwise statistically at par). ns, non-significant (p > 0.05). *Significant at p ≤ 0.05. **Significant at p ≤ 0.01; Other = Tassel + cob + shank + husk; ^$^– sign in post-silking K accumulation represents net loss of K from silking to physiological maturity stage; ^†^HI(K), Harvest Index (Potassium).

At physiological maturity, year 2 produced significantly greater K accumulation in parts of the stalk other than the stem but produced significantly lower grain accumulation of K. The net effect was a reduction in HI(K) for year 2.

At silking, cultivar P3522 exhibited highest K accumulation in leaf and stem but lowest in the tassel. At physiological maturity, it had the greatest accumulation of K in the grain. At silking, Rajkumar had the highest K accumulation in tassel, but the lowest accumulation in the stem and intermediate accumulation in the leaves. P3396 had the lowest K accumulation in the leaves and intermediate accumulation in the stem and tassel. Both of these cultivars had lower grain K accumulation than P3522. At silking, P3522 recorded significantly higher total K accumulation than the other two cultivars; however, there was no significant difference in total K accumulation among cultivars at physiological maturity. At both growth stages, highest value of total K accumulation was recorded in P3522, which may explain its significantly greater loss of K from silking to physiological maturity (indicated by the negative post-silking K accumulation levels).

Application rates of NPK produced significant changes in K accumulation. Application of 125% RDF resulted in the consistently highest K accumulation in different plant parts, both at silking and physiological maturity, and it also resulted in the greatest post-silking K loss. The reduction in K accumulation in various parts and in the total plant by nutrient omission was in the order –N > –K > –P both at silking and physiological maturity stages. The control (–NPK) treatment consistently reduced K accumulation to the greatest extent and reduced post-silking K loss. The 100% RDF treatment produced K accumulation equivalent to the 125% RDF treatment for tassel K accumulation at silking as well as grain K accumulation. It produced the second-highest K accumulation in leaf and stem at silking and in leaf, stem, and other non-grain organs at physiological maturity. Compared with 100% RDF, omitting N lowered K accumulation at silking in the leaf and stem more than omitting K. Similarly, at physiological maturity, omitting N lowered K accumulation more than omitting K in leaves and grain. In the remaining cases, omitting N and K resulted in similar K accumulation. Omitting P resulted in K accumulation similar to omitting K in all but leaves at silking and grain at physiological maturity. The NPK rate at 50% RDF resulted in the lowest post-silking K losses.

### Potassium Remobilization and Its Contribution to Grain Potassium Accumulation

Potassium remobilization efficiency (%) and contribution to grain K (%) as influenced by year, cultivar, and levels of NPK are described in **Table 8**. The year effect on leaf and stalk K remobilization efficiency was non-significant (*p* > 0.05). Cultivar exerted significant influence on leaf and stalk K remobilization efficiency. P3396 had lower leaf remobilization efficiency than the other two cultivars and its leaves acted as a sink rather than a source of K for other organs and did not contribute to grain K (shown by the negative contribution to grain K). Compared with Rajikumar, both P3396 and P3522 had higher percentages of K remobilization from the stalk and associated higher contributions of the stalk to grain K. Levels of N, P, and K affected only stalk K remobilization to the grain, with the greatest remobilization occurring with 125% RDF. Rates of 50 and 75% RDF produced the lowest percent remobilization of K from stalk to grain.

**Table 8:** Potassium remobilization efficiency (%) and contribution to grain K (%) in maize as influenced by year, cultivar, and levels of NPK.

Treatments	K remobilization efficiency		Contribution to grain K by K remobilization	
	Leaf	Stalk	Leaf	Stalk
*Year*				
Year 1	+11.8a	+48.4a	12.2a	151.7a
Year 2	+9.1a	+47.2a	11.9a	184.0a
*Cultivar*				
P3522	+18.2a	+49.7a	17.8a	170.2a
P3396	−9.8b	+50.3a	–5.4b	177.0a
Rajkumar	+23.0a	+43.4b	23.9a	156.3b
*Levels of NPK*				
N_100_P_30_K_30_ (50% RDF)	+3.8a	+36.3a	5.0a	102.4d
N_150_P_45_K_45_ (75% RDF)	+0.9a	+47.2a	0.8a	149.7cd
N_200_P_60_K_60_ (100% RDF)	+3.9a	+47.7a	5.6a	184.6b
N_250_P_75_K_75_ (125% RDF)	+1.3a	+49.9a	3.8a	213.6a
N_300_P_90_K_90_ (150% RDF)	+9.3a	+49.2a	12.5a	173.5b
P_60_K_60_ (100% RDF–N)	+18.7a	+48.3a	23.1a	176.2b
N_200_K_60_ (100% RDF–P)	+21.9a	+50.5a	21.5a	161.9bc
N_200_P_60_ (100% RDF–K)	+19.2a	+51.4a	19.9a	168.2bc
Control (–NPK)	+15.1a	+49.6a	16.6a	180.6b
*Source of variation*				
Year	ns	ns	ns	ns
Cultivar	*	*	*	*
Levels of NPK	ns	ns	ns	*
Year × cultivar	ns	ns	ns	ns
Year × levels of NPK	ns	ns	ns	ns
Cultivar × levels of NPK	ns	ns	ns	*
Year × cultivar × levels of NPK	ns	ns	ns	ns

Year 1 and year 2 represent winter 2012–13 and 2013–14, respectively. Within year, cultivar, and levels of NPK, numbers followed by different letters indicate significant differences at p ≤ 0.05 (otherwise statistically at par). ns, non-significant (p > 0.05). *Significant at p ≤ 0.05, + and − sign represent the situations when the tissue is acting as a source and sink, respectively.

### Grain and Stover Yield

Both year and cultivar effects were found to be non-significant for grain yield and stover yield (**Table 9**). Despite the lack of statistical significance, there was a general trend indicating that the cultivar P3522 produced the highest yield (both grain and stover) followed by P3396 and Rajkumar, respectively. Levels of NPK had significant influence on grain and stover yields. Both grain and stover yields increased significantly (*p* ≤ 0.01) up to 75% RDF, 94.2 and 65.1% more than grain and stover yields under the control (–NPK) plot. Significant yield (both grain and stover) reduction was observed in nutrient omission plots, while the lowest yield was recorded in the control plot. Omission of N caused greater reduction in grain and stover yield, followed by K omission and P omission. When compared with 100% RDF, grain yield reduction with nutrient omission was 43.9% for N omission, 16.8% for P omission, and 26.9% for K omission.

**Table 9:** Grain yield (Mg ha^–1^) and stover yield (Mg ha^–1^) of maize as influenced by year, cultivar, and levels of NPK.

Treatments	Grain yield	Stover yield
*Year*		
Year 1	7.62a	5.83a
Year 2	8.16a	6.06a
*Cultivar*		
P3522	8.34a	6.71a
P3396	7.78a	5.89a
Rajkumar	7.54a	5.24a
*Levels of NPK*		
N_100_P_30_K_30_ (50% RDF)	8.39b	6.13bcd
N_150_P_45_K_45_ (75% RDF)	8.97ab	6.57abc
N_200_P_60_K_60_ (100% RDF)	9.43a	7.25ab
N_250_P_75_K_75_ (125% RDF)	10.37a	7.61a
N_300_P_90_K_90_ (150% RDF)	9.17ab	6.80ab
P_60_K_60_ (100% RDF–N)	5.29d	4.53ef
N_200_K_60_ (100% RDF–P)	7.85bc	5.54cde
N_200_P_60_ (100% RDF–K)	6.89c	5.11def
Control (–NPK)	4.62d	3.98f
*Source of variation*		
Year	ns	ns
Cultivar	ns	ns
Levels of NPK	**	**
Year × cultivar	ns	ns
Year × levels of NPK	ns	ns
Cultivar × levels of NPK	ns	ns
Year × cultivar × levels of NPK	ns	ns

Year 1 and year 2 represent winter 2012–13 and 2013–14, respectively. Within year, cultivar, and levels of NPK, numbers followed by different letters indicate significant differences at p ≤ 0.05 (otherwise statistically at par). ns, non-significant (p > 0.05). **Significant at p ≤ 0.01.

## Discussion

### Partitioning of Dry Matter

**Figure 1:**
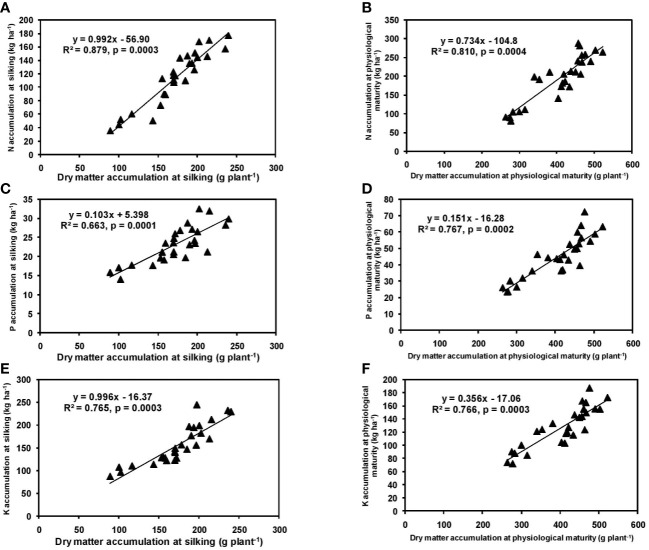
Relation between dry matter accumulation and N **(A, B)**, P **(C, D)**, and K **(E, F)** accumulation at silking, and physiological maturity stage of hybrid maize.

In the present study, significant differences in DMA among cultivars were observed during both silking and physiological maturity stages. The cultivar P3522 accumulated higher total dry matter than P3396 and Rajkumar (**Table 1**). Application of 125% RDF produced highest DMA at both phenophases for all cultivars; however, further increment of NPK did not increase DMA. A linear and positive regression between DMA and NPK accumulation at silking and physiological maturity stages has been indicated by the slopes and intercepts of the respective equations (**Figure 1**), leading to greater DMA with higher NPK rates. Previous studies also reported higher DMA with increased levels of NPK (Juan Valero et al., 2005; Gul et al., 2015). Earlier, Gul et al. (2015) also reported an increase in DMA from 6.6 to 7.6 Mg ha^–1^ at silking stage and from 10.0 to 11.3 Mg ha^–1^ at maturity with an increase in NPK level from 60:40:20 to 90:60:40. They mentioned that higher numbers of leaves and higher LAI with better fertilization might have produced more photosynthetic dry matter resulting in higher DMA. In the present study (**Table 1**), the interaction of cultivar and NPK rates produced significant variation in leaf and stem DMA in cultivars at silking; however, this variation became non-significant (*p* > 0.05) at physiological maturity. This might be attributed to higher post-silking dry matter accumulation in all the tested maize hybrids (Ning et al., 2013).

It is noteworthy that we found very limited information on the difference of DMA among hybrid maize cultivars at different NPK levels; therefore, this study provides useful insights, especially for agronomists and frontline extension professionals.

### Post-Silking Dry Matter Accumulation, Remobilization, and Contribution to Grain Dry Matter

In the present study, cultivar exerted significant influence on PSDMA (**Table 1**), remobilization, and contribution to grain dry matter (**Table 2**). According to Chen et al. (2014), plant cultivars having different traits exhibited variable grain yields and grain N concentration. In our study, PSDMA increased with successive increases in NPK levels up to 125% RDF, and thereafter it declined. Hence, it is clear that higher levels of NPK accumulated more dry matter in vegetative organs (tissues acted as sink), and thereby remobilized more dry matter. In contrast, nutrient omissions lowered the PSDMA resulting in poor dry matter remobilization and the least contribution to grain dry matter. Chen et al. (2015) also demonstrated that the N application rates significantly affected vegetative dry matter remobilization efficiency in maize hybrids, and that remobilization was increased with a decrease in N rates from 240 (5%) to 0 kg Nha^–1^ (16%). Other studies also confirmed considerable increase in stalk dry matter remobilization with greater contribution to grain dry matter at lower NPK rates (Chen et al., 2014). Some investigators mentioned that new maize cultivars accumulate more post-silking dry matter than old cultivars, which may ultimately contribute significantly to higher grain yield (Echarte et al., 2008).

Maize shows an enormous growth rate due to high CO_2_ assimilation through the C_4_ pathway. In order to meet yield potential, the nutrient requirements of maize hybrids per day are high (Nelson, 1968). The level of applied NPK must also be high under conditions with high photosynthetic activity of winter maize (high light intensity, optimum temperature, and absence of water stress) (Mengel and Kirkby, 2006). In the present study, omission of NPK significantly reduced DMA in different plant organs. Reduction of DMA was greater with N omission, followed by K and P omission, respectively. The higher impact of K than P on reduction of DMA was probably due to low K fertility in the study location. Such observations indicate the essentiality of these macronutrients and probable interactions among them for better growth of maize hybrids. The plants with the low N supply accumulate carbohydrate, particularly starch and fructans in leaves and stems, whereas their concentration of crude protein becomes depressed (Ray et al., 2019). This suggests that inadequate N nutrition may have limited the utilization of photosynthates, particularly for the synthesis of organic N compounds. The rest could be stored in the form of starch and fructans. By contrast, adequate supply of N may have augmented plant growth as a result of more photosynthetic surface and leaf area index (LAI), thereby contributing more to DMA (Jasemi et al., 2013).

For optimum vegetative growth, the required N nutrition must also be balanced by the presence of other macronutrients; otherwise, insufficient K (Scherer et al., 1982) and P supply (Fredeen et al., 1989) may restrict vegetative growth in conjunction with greater accumulation of carbohydrates in leaves. Nucleic acid synthesis depends on phosphate while K^+^ concentration in cytosol is the causal factor for protein synthesis (Wyn Jones and Pollard, 1983). Mengel and Kirkby (2006) also suggested that K is important for growth and elongation, probably in its function as an osmoticum, and may react synergistically with indole acetic acid (IAA). However, these results are not always comparable for several reasons (biomass measurements without considering below-ground and dead plant parts, genetic variation, different plant populations and sowing times, different climatic conditions).

### Nutrient Dynamics Within the Maize Plant

In the present study, the overall trend of N accumulation in leaves at two phenophases (silking and physiological maturity) of tested maize cultivars was more or less similar; however, N accumulation was reduced considerably in stems at physiological maturity (**Table 3**), with greater N accumulation in grain, and the trend was grain > leaf > stem > other part of stalk. Thom and Watkin (1978) observed that during the early part of maize growth, the major sink for N is leaves, while grain assumes the role of sink at maturity and contains about 60% of total N. Hanway (1962) also had a similar opinion that N is not generally translocated to grain from other plant parts until the “blister” or “milk” stage of grain development. Kosgey et al. (2013) reported that the N accumulation in leaf was higher during early stages but declined later at kernel filling stage. These researchers also observed that the stalk N accumulation was higher at early kernel filling but declined thereafter and this trend was similar to the present study. Swank et al. (1982) posited that stalk was the key source of N for early kernel filling in irrigated crops. The results presented here revealed that at silking both P and K accumulation were of the order stem > leaf > tassel. At physiological maturity, the trend for P accumulation was grain > leaf > stem > other part of stalk, and that for the K accumulation was stem > grain > leaf > other part of stalk. The justification for variation in N accumulation in different plant parts, as discussed above, might also be applicable for P accumulation during two phenophases; however, the different trend for K was attributed to lower post-silking K accumulation with its contribution to grain K, and higher amount of mobilized K from vegetative parts (tissue acted as source).

### Post-Silking Nutrient Accumulation, Remobilization, and Their Contribution to Grain Nutrients

The PSNA, and remobilization and their (remobilized N) contribution to grain varied significantly with cultivar (**Tables 3** and **4**). The cultivar P3522 had the greatest vegetative N remobilization and contribution to grain N; however, it had the lowest post-silking N accumulation. Borrell et al. (2001) found that the maize hybrids differ in their patterns of PSNA and leaf N remobilization efficiencies. For the present study, tested cultivars P3522, Rajkumar, and P3396 can be designated as low, medium, and high “stay-green” hybrids as per the metrices used by Kosgey et al. (2013). The application of 150% RDF in the present study produced the highest PSNA (**Table 3**). This finding corroborates the result of Chen et al. (2015) who also observed an increasing trend of PSNA with greater N application. In the present study, comparatively higher N remobilization efficiency was exhibited by stalks than leaves, because in few cases leaf tissue acted as a sink rather than a source. The same was the case for its contribution to grain N. It is interesting to note that nutrient omission treatments contributed more to grain N by N remobilization which is being attributed to lesser PSNA at these NPK levels. Furthermore, Chen et al. (2015) concluded that N and genotype are two major factors affecting grain yield and grain N concentration in maize. Moreover, N remobilization from vegetative tissues and post-silking N accumulation contribute to grain N; however, their relative contributions are genotype-speciﬁc, and are affected by the N application rate. In principle, high grain yield is associated with lower grain N concentration due to the dilution effect (Uribelarrea et al., 2007). However, impacts of cultivars on P and K accumulation, remobilization, and contributions were not available in the literature.

The post-silking P accumulation (PSPA), remobilization, and their contribution to grain varied significantly due to cultivar (**Table 5**). The cultivar P3396 recorded the highest PSPA with lower P remobilization and contribution to grain P. Higher leaf and stalk P remobilization with greater contribution to grain P content was recorded in Rajkumar and P3522, respectively. Results of the present study also revealed that successive increase in levels of NPK up to 125% RDF brought about significant increase in PSPA (368% higher than control). The P omission drastically reduced PSPA, and it was statistically at par with the control treatment (–NPK). Furthermore, NPK fertilization brought about significant variations in leaf and stalk P remobilization and their contribution to grain P. Plants in nutrient omission plots as well control plots showed greater vegetative P remobilization and contribution to grain while the control treatment exhibited maximum vegetative P remobilization and contribution to grain.

The tested cultivars exhibited non-significant effect on post-silking K loss, while the variation was significant for K remobilization and contribution to grain K (**Table 8**). Further, levels of NPK influenced post-silking K accumulation significantly. Fertilization with successive increases in NPK levels from 50 to 125% RDF resulted in decreased post-silking K accumulation. Present day, modern maize hybrids are characterized by prolonged photosynthetic activity, delayed leaf senescence, greater post-silking NPK accumulation, and poor remobilization and recycling of leaf NPK during grain-filling. Hence, higher rates of vegetative remobilization and recycling of NPK (accumulated during pre-silking) are not always obligatory for greater accumulation of grain NPK. Since N, P, and K jointly takes part in photosynthesis (Hawkesford et al., 2012), remobilization and recycling of reserved NPK from leaf tissues will affect photosynthesis. Typically, PSKA was very low (<1 kg ha^–1^), but the stalk K remobilization contributed more than 100% to grain K. Contrarily, stalk N and P remobilization and their contribution to grain N and P was quite low. In the present study, such behavior of K can be understood as an “extra remobilization contribution” indicator, and for N and P behavior, it can be assessed as “nutrient remobilization contribution disabled,” to some extent, as suggested by Masclaux-Daubresse and Chardon (2011). Comparatively higher K accumulation was recorded in the leaf and stalk part during silking to physiological maturity, even more than that accumulated in grain, which suggests loss of K from the leaf and stalk part. Earlier studies already proved that leaching (Tukey and Tukey, 1969) and guttation (Lessani and Marschner, 1978) are the two prime causes for such loss of nutrients, including K, from vegetative parts. Leaching loss of nutrients from leaf or stalk depends on several internal (nature or type of plant, age of the plant, and nutrient status in organ) and external (leaching solution, light and temperature, rainfall, greenhouse and field grown plants, etc.) factors. In an earlier study, maize was observed to leach K about two to four times more than tomato (*Solanum lycopersicum* L.) and five to eight times more than sugar beet (*Beta vulgaris* L.) (Tukey, 1958). Due to the hydrophobic nature of young plants, leaves become wet (Linskens, 1952) and thereby K leaches out from the leaf as the crop grows towards physiological maturity (Stenlid, 1958; Tukey, 1958). During grain filling, nutrient requirements for grain are fulfilled either from plant nutrient accumulation from the soil and/or remobilization of nutrient reserve (nutrient accumulated before grain filling). The proportion derived from each fraction depends on the availability of soil N and the extent of remobilization of stored N reserves. The results also implied that N and P taken up during grain filling were the primary sources for grain nutrient concentration. When the amount of N and P accumulated during post-silking stage could not fulfill the grain requirement, then pre-silking accumulated N and P were remobilized. Other investigators similarly observed that under N or P deficiency, more N and P accumulated in vegetative parts during pre-flowering stages were remobilized to maize grains (He et al., 2004; Peng and Li, 2005; Pommel et al., 2006). Moreover, the extent of nutrient (NPK) accumulation at different growth stages is closely related to their roles in plants.

Large amounts of N, P, and K accumulated during pre-silking are remobilized and exported from vegetative tissues, especially from leaves, during grain ﬁlling in tested cultivars. Asynchronous accumulation and remobilization of NPK is closely associated to their individual roles in plant growth. Potassium plays multifarious roles in enzyme activation, transport of nutrients and assimilates in the cytoplasm, and maintenance of cell osmotic potential which supports vigorous vegetative growth (Hawkesford et al., 2012), which might corroborate with higher K accumulation during pre-silking stage. In comparison, N and P requirement is higher for the development of new tissues, both during rapid pre-silking vegetative growth and post-silking grain development stages.

### Maize Yield and Its Relation With Dry Matter, N, P, and K Accumulation at Different Growth Stages

**Figure 2:**
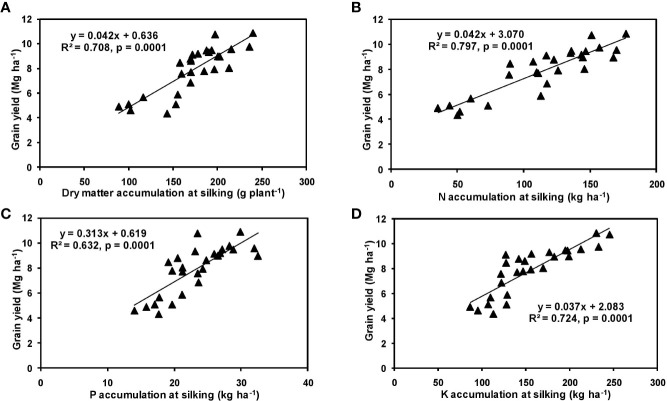
Relation between grain yield and dry matter **(A)**, N **(B)**, P **(C)**, and K **(D)** accumulation of hybrid maize at silking stage.

**Figure 3:**
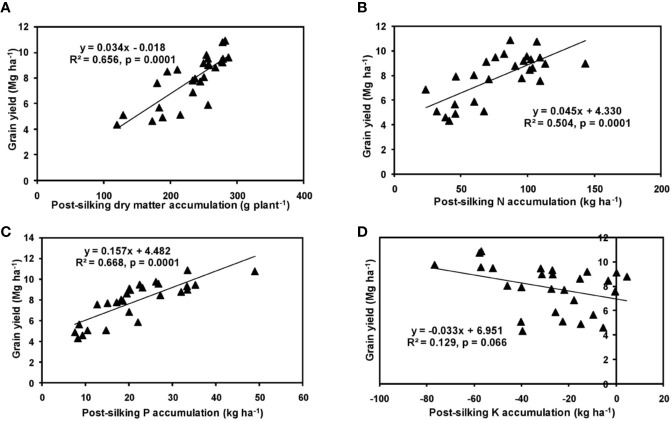
Relation between grain yield and dry matter **(A)**, N **(B)**, P **(C)**, and K **(D)** accumulation of hybrid maize at post-silking stage.

**Figure 4:**
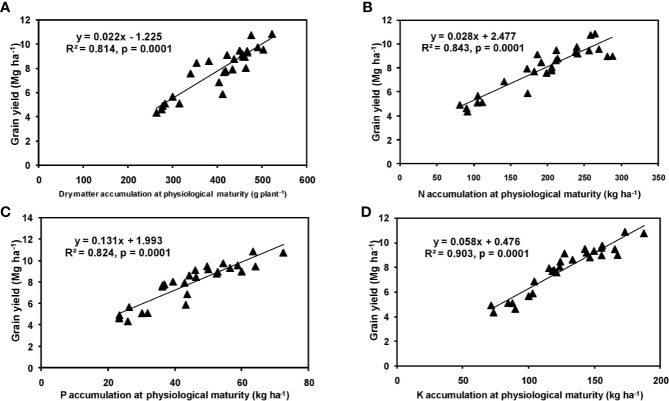
Relation between grain yield and dry matter **(A)**, N **(B)**, P **(C)**, and K **(D)** accumulation of hybrid maize at physiological maturity stage.

The effects of year and cultivar factors on grain and stover yields of tested maize hybrids were found to be non-significant; however, NPK levels had significant effects on them. The grain yields produced at 75% RDF were statistically similar to the yield achieved at higher rates. Both grain and straw yields were found to be increased with increasing N levels up to 125% RDF, and thereafter declined. However, increase in N level from 75 to 150% RDF did not bring about significant variation in grain and straw yields of maize hybrids. Close inspection of the data however reveals that greater dry matter accumulation was achieved at 125% RDF. This result suggests that plants tried to maintain a synchrony between the vegetative and the reproductive stage up to 75% RDF, but failed to maintain the same at 100 and 125% RDF. In case of tested maize hybrids with stay green nature, the formation of new reproductive structures is coupled to the development of vegetative structures, and vegetative growth can therefore not become zero. This coupling causes strong allometry between vegetative and reproductive weight, especially at higher fertilizer doses (100 and 125% RDF). Omission of nutrients reduced grain yields. The grain yield reduction was in the order −N plot > −K plot > −P plot. It clearly depicts the fact that N is the most limiting nutrient for maize followed by K and P in the study location. Greater importance of N fertilization to maize productivity has also been revealed by Liu et al. (2011) and Ray et al. (2018). In the present experiment, grain yield had positive relations with dry matter, N, P, and K accumulation at silking, post-silking, and physiological maturity stages, as indicated by the slopes and intercepts of the linear equations in **Figures 2****–****4**; however, grain yield had the lowest association with post-silking K accumulation. Further inspection of the data reveals that K accumulation at physiological maturity was less than that of silking stage which clearly signifies post-silking K loss, as observed in our study. Moreover, grain K concentration at physiological maturity was largely contributed by K remobilization from leaf and stalk portion. Hence, post-silking K accumulation failed to exert significant impact on grain yield. Positive effects of dry matter (Birch et al., 1999; Bodnár et al., 2018), N (Tsai et al., 1992; Plénet and Lemaire, 1999), P (Wang and Ning, 2019; Ahmed et al., 2020), and K (Bhattacharyya et al., 2018; Ul-Allah et al., 2020) accumulation on grain yield of maize have been well documented by previous researchers across different geographical locations. Findings of our study agree with those outcomes of the previous research. Besides clarifying the dynamics of the macronutrients within the plant parts at various levels of NPK application, this study also highlights the complimentary role of each nutrient in its translocation and remobilization as well as yield building in maize. The necessity of supplying adequate amounts of the major nutrients to stabilize maize yield in the alluvial soils of humid-tropics is further underlined.

## Conclusion

In most of the previous studies, the concentration of N in different parts of the maize plant, its remobilization, post-silking accumulation, and finally its subsequent effect on grain yield was highlighted. The novelty of the present study was assessing the simultaneous effect of all three macronutrients (N, P, and K) on their dynamics in the maize plant and its yield. The data obtained from the study support the following conclusions:

- Highest post-silking DMA and nutrient accumulation were observed in cultivar P3396; however, P3522 produced the highest nutrient remobilization and contribution to grain nutrient accumulation.
- Application of 125% RDF was optimum for post-silking DMA in maize plants. Post-silking N accumulation continued to increase up to application rates of 150% RDF, while post-silking P and K accumulation was optimized at 125% RDF.
- The choice of cultivars had a significant effect on the remobilization efficiency as well as contributions to grain nutrient accumulation for all tested macronutrients (N, P, and K).
- In general, remobilization efficiency as well as contribution to grain nutrient accumulation was maximized at 125% RDF for N and K, but 100% RDF for P (either significantly or at par with other rates) for plots receiving nutrients; however, for all tested cultivars, nutrient remobilization and contribution to grain nutrient accumulation was highest under nutrient-omission plots and absolute control plots.
- Omission of N had the highest negative impact on post-silking as well as total DMA and nutrient accumulation in all tested cultivars, followed by P and K omission.
- Application of 75% RDF brought significant improvement in both grain and stover yields. Further increases in NPK application rates did not increase grain yields. Hence, 150 kg N, 45 kg P_2_O_5_, and 45 kg K_2_O ha^−1^ was found sufficient to achieve attainable yield at the study location.
- The cultivar P3522 produced higher yield than P3396 and Rajkumar, irrespective of fertilizer doses, although the difference was statistically insignificant and therefore suggests further study.
- The dry matter accumulation and NPK accumulation patterns at different physiological growth stages of maize vary with changes in growing season, cultivar, and NPK levels, and those accumulations are positively correlated with grain yield.

## Data Availability Statement

All datasets generated for this study are included in the article/**Supplementary Material**.

## Author Contributions

KR, HB, and SD conceptualized the work. Field experiment and laboratory analysis was carried out by KR, SS, and HB. Methodology was framed by HB, SD, TM, VS, and KM. The work was supervised by HB, SD, and KM. Original draft was prepared by KR, SS, HB, and SD. Review and editing part was taken care of by HB, SD, TM, VS, and KM.

## Funding

The work was supported by Research and Communication Fund of African Plant Nutrition Institute, Benguérir, Morocco.

## Conflict of Interest

The research was conducted in the absence of any commercial or financial relationships that could be construed as a potential conflict of interest.

## Supplementary Material

S1 File (Table A): Initial physico-chemical properties of the experimental soil (0–30 cm depth).
