# Supplementary material for: Macronutrient Management Effects on Nutrient Accumulation, Partitioning, Remobilization, and Yield of Hybrid Maize Cultivars
Source: Front Plant Sci. 2020 Sep 2;11:1307. doi: 10.3389/fpls.2020.01307 (PMC7492750; doi:10.3389/fpls.2020.01307)
Supplement: S1 File — (Table A): Initial physico-chemical properties of the experimental soil (0–30 cm depth). [file Table_1.docx]

**Table A** Initial physico-chemical properties of the experimental soil (0-30 cm depth)

| Parameter | Result | | Methodology | Citation | Equipment used |
| --- | --- | --- | --- | --- | --- |
|  | Winter Year 1 | Winter Year 2 |  |  |  |
| Mechanical composition | | | | | |
| a) Sand (%) | 36.8 | 35.5 | Hydrometer method | Bouyoucos (1962) | Hydrometer |
| b) Silt (%) | 28.0 | 27.5 |  |  |  |
| c) Clay (%) | 35.2 | 37.0 |  |  |  |
| Soil texture | Clay-loam | Clay-loam | Textural triangular method | Brady and Weil (1996) | - |
| pH | 7.31 | 6.88 | (in 1:2.5:: Soil : Water) | Jackson (1967) | μ-processor based pH-EC-Ion meter |
| EC (dS m^‒1^) | 0.30 | 0.15 | (in 1:2.5:: Soil : Water) | Jackson (1967) |  |
| Organic carbon (%) | 0.66 | 0.54 | Wet oxidation method | Jackson (1973) | - |
| Available N (kg ha^‒1^) | 215.2 | 172.4 | Hot alkaline KMnO_4_ Method | Subbiah and Asija (1956) | Kjeldahl apparatus |
| Available P (kg ha^‒1^) | 41.6 | 53.9 | 0.5 M NaHCO_3_ extract | Olsen et al. (1954) | Spectrophotometer |
| Available K (kg ha^‒1^) | 186.4 | 190.9 | Neutral N NH_4_OAc extract | Hanway and Heidel (1952) | Flame photometer |

Year 1 and year 2 represents winter 2012-13 and 2013-14, respectively.

**References**

Bouyoucos GJ (1962). Hydrometer method improved for making particle size analysis of soils. Agron. J. 54: 464-465.

Brady NC, Weil RR (1996) The Nature and Properties of Soils, 11th edition. Upper Saddle River, N.J., Prentice Hall.

Hanway JJ, Heidel H (1952) Soil analysis methods as used in Iowa State College Soil Testing Laboratory. Iowa Agric. 57: 1-13.

JacksonML(1967) Soil chemical analysis. Prentice Hall of India Pvt. Ltd, New Delhi, India.

Jackson ML (1973) Soil chemical analysis. Prentice Hall of India Pvt. Ltd, New Delhi, India.

Subbiah B. Asija, GL (1956) A rapid procedure for the estimation of available N in soils. Current Sci. 25: 259-260.

Olsen SR, Cole CV, Watanale FS, Dean LA (1954) Estimation of available phosphorus in phosphorus in soils by extraction with sodium bicarbonate. Washington D.C.: United States Department of Agriculture. Circular 393.
